# Pregnancy, Labor, and Delivery after Ebola Virus Disease and Implications for Infection Control in Obstetric Services, United States

**DOI:** 10.3201/eid2207.160269

**Published:** 2016-07

**Authors:** Amanda Kamali, Denise J. Jamieson, Julius Kpaduwa, Sarah Schrier, Moon Kim, Nicole M. Green, Ute Ströher, Atis Muehlenbachs, Michael Bell, Pierre E. Rollin, Laurene Mascola

**Affiliations:** Centers for Disease Control and Prevention, Atlanta, Georgia, USA (A. Kamali, D.J. Jamieson, U. Ströher, A. Muehlenbachs, M. Bell, P.E. Rollin);; Los Angeles County Department of Public Health, Los Angeles, California, USA (A. Kamali, M. Kim, N.M. Green, L. Mascola);; Greater El Monte Community Hospital, South El Monte, California, USA (J. Kpaduwa, S. Schrier)

**Keywords:** Ebola virus disease, Ebola virus, EBOV, obstetrics, obstetric services, infection control, standard precautions, personal protective equipment, neonate, survivor, viruses, pregnancy, United States

## Abstract

Many of the survivors of the 2014–2015 epidemic of Ebola virus disease (EVD) in West Africa were women of childbearing age. Limited clinical and laboratory data exist that describe these women’s pregnancies and outcomes. We report the case of an EVD survivor who became pregnant and delivered her child in the United States, and we discuss implications of this case for infection control practices in obstetric services. Hospitals in the United States must be prepared to care for EVD survivors.

## Abstract 2

Women who become pregnant after recovery pose little risk for transmitting the virus to the baby or others.

The 2014–2015 epidemic of Ebola virus disease (EVD), which was centered in West Africa, is the largest EVD epidemic in history. Vertical transmission of Ebola virus from mother to fetus can occur during acute Ebola infection, leading to intrauterine fetal death, stillbirth, or neonatal death (*1*–*5*). Little is known about the risk for vertical transmission of Ebola virus from women to their neonates outside of the acute infectious period. Ebola virus (EBOV) has been found in breast milk during acute disease (*6*), and a study documenting 2 discordant mother–child pairs postulated that breast feeding of 1 infant may have led to infection of the infant (*7*). EBOV has been found in immune-privileged sites, ocular fluid and semen, many months after onset of infection (*8*–*13*), and it is possible that other immune-privileged sites, such as the central nervous system (CNS), may also contain EBOV many months after onset of infection. In addition, acutely infected pregnant women have had high amounts of Ebola viral nucleic acid persist in the amniotic fluid following clearance of viremia; however, it is not known whether this amniotic fluid is infectious (*2*). Some theoretical concern exists that during labor and delivery or obstetric anesthetic procedures (e.g., spinal anesthesia), contact with products of conception or cerebrospinal fluid from EVD survivors may pose an infectious risk (*6*,*14*–*18*).

As of March 9, 2016, an estimated 17,323 persons worldwide have survived EVD, and among them are ≈5,000 women of childbearing age (*19*). Survivors will require medical care for routine illnesses, surgical services, dental work, and management of disease sequelae (*20*,*21*). In addition, many of the female survivors who are of reproductive age will require obstetric care. Some of these survivors may come to the United States, and hospitals and healthcare workers must be prepared to provide care in a manner that promotes patient dignity and comfort, prevents stigmatization, and ensures that all patients receive appropriate, high-quality medical care (*22*–*24*). However, limited preparations have been made for follow-up care for EVD survivors, including those needing obstetric care, and to minimize possible stigma and fear. We describe the case of an EVD survivor who delivered a healthy neonate in a community hospital in the United States 14 months after acute EBOV infection, and we discuss the implications of the findings from this case for infection control in obstetric services.

## Clinical Course

### Ebola Virus Disease Course

A 29-year-old physician from West Africa became ill with EVD in late July 2014. She had contracted the virus from an EVD patient whom she had cared for from July 20th until his death on July 25. On July 29, the woman began feeling unwell, noting arthralgia and myalgia, which she self-treated with antimalarial medications. On August 1, she had fever, and on August 3, she began vomiting and had diarrhea. The woman was admitted to an Ebola treatment center (ETC) and isolated after results of an EBOV real-time reverse transcription PCR (rRT-PCR) were positive for EBOV RNA (cycle threshold unknown). According to the woman, she spent 13 days in the ETC, where she was treated with oral rehydration fluids, acetaminophen, and a second course of antimalarial medications. She was discharged from the ETC on August 16, after showing negative results on 2 EBOV rRT-PCRs. After her recovery, the woman noted some fatigue, anorexia, arthralgia, and alopecia; she did not report any sleep disturbances, headaches, or vision problems. Symptoms resolved 2–3 months later.

### Pregnancy, Labor, and Delivery

Eight months before her EVD diagnosis, the patient had had a spontaneous abortion at 10 weeks’ gestation. In January 2015, twenty-two weeks after her last negative EBOV rRT-PCR, she became pregnant again. For this second pregnancy, the estimated date of delivery was established on the basis of an 11.5-week ultrasound that was consistent with the patient’s last menstrual period. The patient received routine prenatal care in West Africa, and at 25 weeks’ gestation, she traveled to Kern County, California, USA, and a detailed anatomy ultrasound was performed in Los Angeles County, California, and demonstrated normal fetal development.

The hospital identified staff members who were willing to assist during labor and delivery for the patient, and at 40 weeks and 1 day of gestation, labor was induced to ensure that those staff members were present. The patient was given 2 vaginal doses of misoprostol, and oxytocin was administered, and labor progressed normally. The patient was given epidural anesthesia for pain control and had a normal vaginal delivery of a female neonate (weight 4,128 g) with Apgar scores of 8 and 9 at 1 and 5 min of age, respectively. The patient had a second-degree perineal laceration, which was repaired.

The patient and her neonate were discharged from the hospital at 36 h postpartum. They returned for routine follow-up 7 days postpartum and were monitored for 6 weeks following delivery, after which they traveled home to West Africa.

### Infection Control and Personal Protective Equipment, Public Health Response

Two weeks before the patient’s delivery date, her US obstetrician contacted the California Department of Public Health (DPH; Richmond, CA, USA) and the Centers for Disease Control and Prevention (CDC; Atlanta, GA, USA) to determine if there were any special precautions needed for infection control; the California DPH notified the Los Angeles County DPH (Los Angeles, CA, USA). Because the patient was healthy and had fully recovered from EVD ≈4 months before becoming pregnant, all public health agencies agreed that she presented an extremely low risk for transmission of Ebola virus. Nevertheless, it was deemed appropriate that public health officials play an active role assessing and guiding management of the patient. The Los Angeles County DPH and CDC collaborated with the hospital’s healthcare providers, nursing directors, laboratory director, environmental services staff, anesthesiologists, and hospital administration to address concerns and review the care plan, including plans for any complications, such as the need for cesarean delivery or the development of peripartum fever.

Hospital infection control procedures were reviewed in person with hospital staff. In review of these policies, no additional precautions were recommended above the standard precautions and policies currently used for all deliveries at the hospital. Several hospital staff members not directly involved in patient care expressed discomfort about working while an EVD survivor was admitted. To reassure these staff members, the patient was kept in 1 room during labor and delivery and after delivery. No changes were made to the policies for environmental cleaning or waste disposal.

Hospital staff raised concerns about the possibility of EBOV being harbored in immune-privileged sites (e.g., cerebrospinal fluid) in EVD survivors and, thus, expressed their concerns about a theoretical risk for EBOV transmission (*6*,*14*–*17*). This patient did not show signs or symptoms of CNS involvement during her acute illness or during her pregnancy, which likely indicated a decreased risk of any latent EBOV reservoir in her CNS; thus, it was considered likely that epidural or spinal anesthesia for this patient would not pose an infectious risk to staff. Hospital staff also noted the often imperfect adherence to use of personal protective equipment (PPE) during labor and delivery; thus, they voiced concern over this patient’s history of EVD because large volumes of blood and amniotic fluid are often encountered in typical, uncomplicated vaginal deliveries (*25*). As a result of these concerns, many discussions were held regarding what PPE should be used during labor and delivery. Standard precautions should always be applied in all medical settings, including labor and delivery; however, neither CDC nor the American College of Obstetricians and Gynecologists had tailored recommendations for PPE specifically for vaginal or cesarean deliveries for any patients. Thus, CDC and Los Angeles County DPH developed a preliminary set of recommendations for the patient’s providers regarding the use of PPE (Tables 1, 2) during and after labor and delivery to ensure that standard precautions were implemented. These PPE recommendations were discussed with the providers in the days before the delivery, and staff members were able to ask for clarification and ensure that materials were readily available. These PPE recommendations did not differ from standard precautions, but they explicitly discussed which PPE to use for casual contact, vaginal examinations, labor and delivery, anesthesia, and postpartum care. Routine hand hygiene, the use of barriers for mucous membrane protection, and the use of double gloves for procedures that involve sharps were emphasized.

**Table 1:** Recommendations for use of personal protective equipment by healthcare workers during labor and delivery for a woman who became pregnant after surviving Ebola virus disease, United States, 2015*

Potential exposure	Personal protective equipment						
	Face mask	Face shield	Gown				Fluid-resistant, midcalf boot covers
			Isolation	Fluid-resistant or impermeable†	Gloves		
					Single	Double	
Casual contact with patient							
Performing duties for patient with intact membranes (e.g., delivering food or water, talking with patient, adjusting external monitors)	No	No	No	No	No	No	No
Performing duties for patient with ruptured membranes; no touching of patient or bedding	No	No	No	No	No	No	No
Noncasual contact with patient							
Touching patient with ruptured membranes or bedding of patient with ruptured membranes	No	No	Yes	No	Yes	No	No
Administering epidural	Yes	Yes	Yes	No	No	Yes	Yes‡
Performing vaginal examination	Yes	Yes	No	Yes	Yes	No	Yes‡
Performing obstetric procedures§	Yes	Yes	No	Yes	No	Yes	Yes

*These personal protective equipment recommendations were developed for this particular patient and do not represent a formal recommendation. †Impermeable indicates that the material and construction have demonstrated resistance to synthetic blood and simulated bloodborne pathogens; fluid-resistant indicates demonstrated resistance to water (http://www.cdc.gov/niosh/npptl/topics/protectiveclothing/default.html). ‡To be used if membranes were ruptured. §Procedures include placement of fetal scalp electrode or intrauterine pressure catheter; manual removal of placenta; bimanual massage of uterus.

**Table 2:** Recommendations for use of personal protective equipment by healthcare workers during postpartum care of a woman who became pregnant after surviving Ebola virus disease and during care of her neonate, United States, 2015*

Level of care	Face mask	Face shield	Gown					Fluid-resistant, midcalf boot covers
			Isolation	Fluid-resistant or impermeable†		Gloves		
						Single	Double	
While caring for mother								
Before bedding/gown change	Yes	Yes	No	Yes		Yes	No	Yes
After bedding/gown change (vaginal exam, perineal care)	No, unless splash likely	No, unless splash likely	Yes	No		Yes	No	No
While caring for neonate								
Before bathing	Yes	Yes	No	Yes		Yes	No	Yes
After bathing	No	No	No	No		Yes‡	No	No

*These personal protective equipment recommendations were developed for this particular patient and do not represent a formal recommendation. †Impermeable indicates that the material and construction have demonstrated resistance to synthetic blood and simulated bloodborne pathogens; fluid-resistant indicates demonstrated resistance to water (http://www.cdc.gov/niosh/npptl/topics/protectiveclothing/default.html). ‡To be used if exposure to fluids is likely.

### Laboratory Assessment

One week before delivery, EBOV rRT-PCR testing was performed on the patient’s blood by the Los Angeles County DPH laboratory and the CDC Viral Special Pathogens Branch; both results were negative. As expected, Ebola serum antibodies were detected by ELISA (IgG >1:1600, IgM negative).

After obtaining written informed consent from the patient, healthcare staff obtained the following during and after delivery: vaginal secretions, amniotic fluid (vaginal pool), cord blood, placenta, umbilical cord, breast milk (colostrum collected 16 h after delivery), and oral and ear swab samples from the neonate. Cord blood, colostrum, amniotic fluid, and swab samples were kept refrigerated until processed or frozen on dry ice for shipment to CDC. A placental sample was frozen in a sterile specimen cup and samples of placenta and umbilical cord were placed in buffered formalin and shipped at room temperature to CDC. EBOV rRT-PCR testing was performed on all of these specimens at the Los Angeles County DPH and CDC laboratories by using assays specific for nucleoprotein and viral protein 40 genes.

Placenta, amniotic fluid, and cord blood samples and ear and oral swab samples from the neonate were negative by EBOV rRT-PCR. Attempts were made to recover virus from placenta, amniotic fluid, cord blood, and colostrum at CDC, but no virus was recovered (Table 3). Amniotic fluid, cord blood, and colostrum were tested by ELISA for IgM and IgG against Ebola virus antigens (*26*). Cord blood was negative for IgM and had an IgG titer of >1:1600. Amniotic fluid and colostrum were negative for IgM and IgG. The placenta and umbilical cord were histologically normal, and no Ebola virus antigen was detected by immunohistochemistry (*27*), including in maternal and fetal endothelial cells and leukocytes.

**Table 3:** Laboratory test results for a woman who became pregnant after surviving Ebola virus disease and for her neonate, United States, 2015*

Source	Time of sample collection	rRT-PCR	Ebola antibodies	Immunohistochemistry
Maternal blood	1 week before delivery	Negative	IgG (1:1,600); IgM not detected	NA
Cord blood	At delivery	Negative	IgG (1:1,600); IgM not detected	NA
Amniotic fluid	At delivery	Negative	IgG; IgM not detected	NA
Vaginal swab sample	At delivery	Negative	NA	NA
Neonate ear swab sample	At delivery	Negative	NA	NA
Neonate oral swab sample	At delivery	Negative	NA	NA
Placenta	At delivery	Negative	NA	Negative for Ebola antigen
Umbilical cord	At delivery	NA	NA	Negative for Ebola antigen
Colostrum	1 day after delivery	Negative	IgG and IgM not detected	NA

*NA, not applicable; rRT-PCR, real-time reverse transcription PCR.

## Conclusions

We describe the delivery of a healthy baby to an EVD survivor who became pregnant 22 weeks after clearance of viremia and resolution of post-EVD sequelae (i.e., fatigue, anorexia, arthralgia). At 6 weeks follow-up, before returning to West Africa, the mother and baby were doing well. Given that the mother did not exhibit any signs or symptoms of post-EVD sequelae during her pregnancy, we did not expect to find any EBOV by rRT-PCR in any specimens obtained, and none was detected. It is somewhat surprising that we did not detect Ebola IgG in the colostrum; however, studies of antibodies for other infections have found that levels of IgG and IgM in colostrum are much lower than in serum (*28*), and this might also be true for antibodies against EBOV.

Although we did not detect EBOV RNA in this patient during pregnancy, women who are pregnant during acute EBOV infection usually transmit virus to the fetus and may pose an infectious risk to healthcare providers and others during delivery or abortion (*3*). EBOV can readily cross the placenta, and pathologic examination of placental tissues of patients with confirmed EVD have demonstrated EBOV antigen in the trophoblasts, syncytiotrophoblasts, and circulating maternal macrophages (*4*). EBOV RNA has been demonstrated in amniotic fluid; fetal meconium; vaginal secretions; umbilical cord; buccal swab samples from neonates; and peripheral blood samples from neonates, including those of mothers with cleared viremia (*29*,*30*).

The immune effects of pregnancy in the context of EVD have not been well documented (*3*); however, alterations in the immune system do occur during pregnancy (*31*), which during acute EBOV infection likely increases the risk for a poor outcome, including spontaneous abortion and neonatal death. Unlike the CNS, eye, and male testis, the genital tract of a nongravid female is not traditionally considered an immune-privileged site (*32*–*34*). Laboratory data that demonstrate the absence of EBOV or the presence of antibodies in post-EVD pregnancies are lacking; however, on the basis of epidemiological evidence in the field of multiple uneventful deliveries in West Africa and of the laboratory-analyzed case reported here, no evidence currently exists that Ebola virus can persist in the female genital tract. Any perceived risk must be mitigated to ensure that patients are not stigmatized and receive appropriate care. The authors concur with current guidelines by the World Health Organization, which state that women who have recovered from EVD are not infectious and should receive routine prenatal care, and their labor and delivery should be performed using standard PPE for protection against blood and body fluids (*35*).

The normal pregnancy for the patient described in this study and her delivery of a healthy neonate offer reassurance that women who become pregnant after recovery from EVD pose little risk for transmission of EBOV to the baby or others. Many more EVD survivors will become pregnant and deliver, and some may do so in the United States. Many other survivors will require routine medical care, including care for post-EVD syndrome. Lessons learned from this patient, specifically those addressing concerns about potential risks for virus transmission, may be applied to future patients. However, each survivor who seeks medical care will likely need to be assessed individually to determine possible risks for transmitting virus (*16*,*18*). Over the course of the public health involvement in this case, it became evident that, although standard precautions should routinely be used in all labor and delivery settings, written guidelines for labor and delivery may be useful, given the heightened concern for a theoretical disease transmission risk. We hope that the preliminary recommendations for PPE use during labor and delivery in the case discussed here will provide a template for other professional organizations to create guidelines for use in all labor and delivery settings.
